# 4-(4-Diethyl­amino-2-hydroxy­benzyl­idene­ammonio)-3-methyl­benzene­sulfonate dihydrate

**DOI:** 10.1107/S1600536809035090

**Published:** 2009-09-05

**Authors:** Wei Zhang, Yuan-Tao Chen

**Affiliations:** a Department of Chemistry, Qinghai Normal University, Xining 810008, People’s Republic of China

## Abstract

In the crystal of the title compound, C_18_H_22_N_2_O_4_S·2H_2_O, mol­ecules are linked into a one-dimensional chain structure by C—H⋯O, N—H⋯O, O—H⋯O and O—H⋯N hydrogen bonds.

## Related literature

For the biological and pharmacological activities of Schiff base compounds, see: Bu *et al.* (2001 ▶); Ranford *et al.* (1998 ▶).
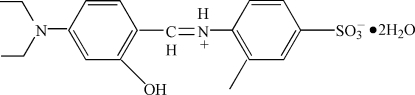


## Experimental

### 

#### Crystal data

C_18_H_22_N_2_O_4_S·2H_2_O
*M* *_r_* = 398.47
Monoclinic, 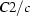

*a* = 21.690 (3) Å
*b* = 11.4142 (17) Å
*c* = 16.639 (2) Å
β = 112.459 (2)°
*V* = 3806.9 (9) Å^3^
*Z* = 8
Mo *K*α radiation
μ = 0.21 mm^−1^
*T* = 273 K
0.19 × 0.16 × 0.06 mm

#### Data collection

Bruker SMART CCD area-detector diffractometer
Absorption correction: multi-scan (*SADABS*; Sheldrick, 1996 ▶) *T*_min_ = 0.962, *T*_max_ = 0.988
9821 measured reflections
3379 independent reflections
2910 reflections with *I* > 2σ(*I*)
*R*_int_ = 0.026

#### Refinement

*R*[*F* ^2^ > 2σ(*F* ^2^)] = 0.054
*wR*(*F* ^2^) = 0.171
*S* = 1.05
3379 reflections
246 parameters
H-atom parameters constrained
Δρ_max_ = 0.73 e Å^−3^
Δρ_min_ = −0.41 e Å^−3^

### 

Data collection: *SMART* (Bruker, 2000 ▶); cell refinement: *SAINT* (Bruker, 2000 ▶); data reduction: *SAINT*; program(s) used to solve structure: *SHELXS97* (Sheldrick, 2008 ▶); program(s) used to refine structure: *SHELXL97* (Sheldrick, 2008 ▶); molecular graphics: *SHELXTL* (Sheldrick, 2008 ▶); software used to prepare material for publication: *SHELXTL*.

## Supplementary Material

Crystal structure: contains datablocks global, I. DOI: 10.1107/S1600536809035090/at2871sup1.cif

Structure factors: contains datablocks I. DOI: 10.1107/S1600536809035090/at2871Isup2.hkl

Additional supplementary materials:  crystallographic information; 3D view; checkCIF report

## Figures and Tables

**Table 1:** Hydrogen-bond geometry (Å, °)

*D*—H⋯*A*	*D*—H	H⋯*A*	*D*⋯*A*	*D*—H⋯*A*
N1—H1⋯O6	0.86	2.10	2.723 (3)	129
N1—H1⋯O2^i^	0.86	2.58	3.160 (3)	125
O6—H6⋯O2^ii^	0.82	1.91	2.709 (3)	166
O4—H16⋯O3^i^	0.85	2.00	2.828 (5)	166
O5—H18⋯N1^iii^	0.85	2.51	3.35 (3)	174
C2—H2⋯O3	0.93	2.56	2.915 (4)	103
C5—H5⋯O1^iv^	0.93	2.55	3.392 (3)	150
C7—H7*B*⋯O2^i^	0.96	2.44	3.325 (3)	154
C8—H8⋯O1^iv^	0.93	2.43	3.353 (3)	172
C15—H15*B*⋯O3^ii^	0.97	2.50	3.444 (4)	166
C16—H16*C*⋯O5^v^	0.96	2.51	3.42 (3)	160

Symmetry codes: (i) 
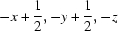
; (ii) 
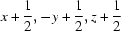
; (iii) 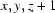; (iv) 
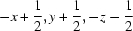
; (v) 
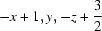
.

**Fig. 1.**
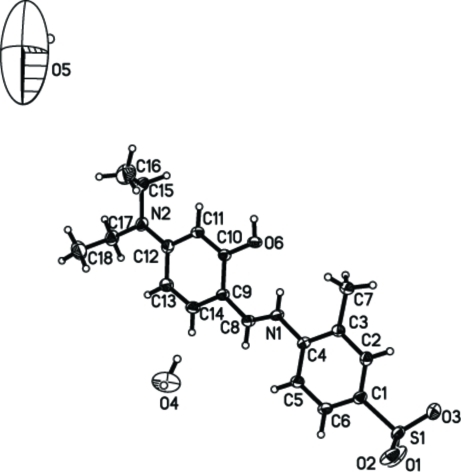
The molecular structure of (I) showing 30% displacement ellipsoids.

## supplementary crystallographic information

### Comment

Schiff bases play an important role in the field of bioinorganic chemistry because they have remarkable wide biological and pharmacological activities, such as antitumor, antidiabetic, antitubercular activities [Ranford, *et al.*, 1998; Bu, *et al.*, 2001]. Therefore, investigating the synthesis and proper ties of hydrazone of these compounds seems to be a very interesting problem. as one part of our systematic work, In this paper, we report on the synthesis and crystal structure of the title compound, (I), (Fig. 1).

The dihedral angle between the aromatic ring planes (C1–C6) and (C9–C14) is 5.70 (12)°, showing that the whole compound is not a plane molecule. The bond distances of C8—N1 [1.311 (3) Å], S1—O2 [1.449 (2) Å] and S1—O3 [1.426 (3) Å] are consistent with the carbon-nitrogen and sulfur-oxygen double-bond lengths, respectively. In the crystal packing, the molecules form a one-dimensional chain structure by C—H···O, N—H···O, O—H···O and O—H···N hydrogen bonds (Table 1).

### Experimental

The solution of 1.0 mmol 4-(diethylamino)salicylaldehyde was added to a solution of 1.0 mmol 3-methyl-benzenesulfonic acid in 5 ml ethanol at room temperature. The mixture was refluxed for 4 h with stirring, then the resulting precipitate was filtered, washed, and dried *in vacuo* over P_4_O_10_ for 48 h. Single crystals suitable for X-ray structural analysis was obtained by slowly evaporating from methanol at room temperature.

### Refinement

All H atoms were included in the refinement at calculated positions, in the riding-model approximation, with C–H distances of 0.93 (ArH), 0.98 (CH_3_), 0.97Å (CH_2_), N–H = 0.86 Å (NH), O–H = 0.82 Å (OH) and 0.85 Å (H_2_O) . The isotropic displacement parameters for all H atoms were set equal to 1.2 or 1.5*U*_eq_ of the carrier atom.

### Crystal data

**Table:**

C_18_H_22_N_2_O_4_S·2H_2_O	*F*(000) = 1696
*M**_r_* = 398.47	*D*_x_ = 1.390 Mg m^−^^3^
Monoclinic, *C*2/*c*	Mo *K*α radiation, λ = 0.71073 Å
Hall symbol: -C 2yc	Cell parameters from 4718 reflections
*a* = 21.690 (3) Å	θ = 2.2–28.4°
*b* = 11.4142 (17) Å	µ = 0.21 mm^−^^1^
*c* = 16.639 (2) Å	*T* = 273 K
β = 112.459 (2)°	Block, colourless
*V* = 3806.9 (9) Å^3^	0.19 × 0.16 × 0.06 mm
*Z* = 8	

### Data collection

**Table:**

Bruker SMART CCD area-detector diffractometer	3379 independent reflections
Radiation source: fine-focus sealed tube	2910 reflections with *I* > 2σ(*I*)
graphite	*R*_int_ = 0.026
φ and ω scans	θ_max_ = 25.1°, θ_min_ = 2.0°
Absorption correction: multi-scan (*SADABS*; Sheldrick, 1996)	*h* = −11→25
*T*_min_ = 0.962, *T*_max_ = 0.988	*k* = −13→13
9821 measured reflections	*l* = −19→19

### Refinement

**Table:**

Refinement on *F*^2^	Secondary atom site location: difference Fourier map
Least-squares matrix: full	Hydrogen site location: inferred from neighbouring sites
*R*[*F*^2^ > 2σ(*F*^2^)] = 0.054	H-atom parameters constrained
*wR*(*F*^2^) = 0.171	*w* = 1/[σ^2^(*F*_o_^2^) + (0.1015*P*)^2^ + 5.082*P*] where *P* = (*F*_o_^2^ + 2*F*_c_^2^)/3
*S* = 1.05	(Δ/σ)_max_ < 0.001
3379 reflections	Δρ_max_ = 0.73 e Å^−^^3^
246 parameters	Δρ_min_ = −0.41 e Å^−^^3^
0 restraints	Extinction correction: *SHELXL97* (Sheldrick, 2008), Fc^*^=kFc[1+0.001xFc^2^λ^3^/sin(2θ)]^-1/4^
Primary atom site location: structure-invariant direct methods	Extinction coefficient: 0.0019 (5)

### Special details

**Table:**

Geometry. All e.s.d.'s (except the e.s.d. in the dihedral angle between two l.s. planes) are estimated using the full covariance matrix. The cell e.s.d.'s are taken into account individually in the estimation of e.s.d.'s in distances, angles and torsion angles; correlations between e.s.d.'s in cell parameters are only used when they are defined by crystal symmetry. An approximate (isotropic) treatment of cell e.s.d.'s is used for estimating e.s.d.'s involving l.s. planes.
Refinement. Refinement of *F*^2^ against ALL reflections. The weighted *R*-factor *wR* and goodness of fit *S* are based on *F*^2^, conventional *R*-factors *R* are based on *F*, with *F* set to zero for negative *F*^2^. The threshold expression of *F*^2^ > σ(*F*^2^) is used only for calculating *R*-factors(gt) *etc*. and is not relevant to the choice of reflections for refinement. *R*-factors based on *F*^2^ are statistically about twice as large as those based on *F*, and *R*- factors based on ALL data will be even larger.

### Fractional atomic coordinates and isotropic or equivalent isotropic displacement parameters (Å^2^)

**Table:**

	*x*	*y*	*z*	*U*_iso_*/*U*_eq_	
S1	0.18156 (3)	0.09473 (5)	−0.21298 (4)	0.0405 (3)	
O1	0.17722 (12)	0.1276 (2)	−0.29916 (14)	0.0750 (8)	
O2	0.11837 (9)	0.1127 (2)	−0.20400 (13)	0.0573 (6)	
O3	0.20786 (13)	−0.0203 (2)	−0.1892 (2)	0.0980 (11)	
O4	0.18994 (19)	0.6795 (4)	0.0958 (2)	0.1365 (14)	
H16	0.2252	0.6420	0.1254	0.205*	
H15	0.1630	0.6669	0.1209	0.205*	
O5	0.276 (2)	0.6202 (16)	1.001 (2)	0.81 (3)	
H18	0.3031	0.5664	1.0016	1.213*	
H17	0.2582	0.6426	0.9487	1.213*	
O6	0.50747 (8)	0.45092 (15)	0.15864 (11)	0.0392 (4)	
H6	0.5392	0.4405	0.2047	0.059*	
N1	0.39104 (9)	0.41470 (17)	0.02004 (12)	0.0332 (5)	
H1	0.4154	0.3891	0.0709	0.040*	
N2	0.60480 (10)	0.83477 (18)	0.19161 (14)	0.0404 (5)	
C1	0.24039 (11)	0.1925 (2)	−0.13942 (15)	0.0343 (5)	
C2	0.28796 (12)	0.1511 (2)	−0.06245 (15)	0.0345 (5)	
H2	0.2863	0.0733	−0.0468	0.041*	
C3	0.33840 (11)	0.2239 (2)	−0.00783 (14)	0.0306 (5)	
C4	0.33861 (11)	0.3409 (2)	−0.03262 (14)	0.0303 (5)	
C5	0.28903 (12)	0.3837 (2)	−0.10848 (16)	0.0387 (6)	
H5	0.2886	0.4624	−0.1230	0.046*	
C6	0.24063 (12)	0.3090 (2)	−0.16183 (16)	0.0412 (6)	
H6A	0.2081	0.3371	−0.2129	0.049*	
C7	0.39164 (13)	0.1746 (2)	0.07302 (16)	0.0442 (6)	
H7A	0.3837	0.0925	0.0774	0.066*	
H7B	0.3905	0.2145	0.1232	0.066*	
H7C	0.4346	0.1853	0.0700	0.066*	
C8	0.40592 (11)	0.5183 (2)	−0.00150 (15)	0.0338 (5)	
H8	0.3790	0.5459	−0.0563	0.041*	
C9	0.45786 (11)	0.59191 (19)	0.04893 (15)	0.0322 (5)	
C10	0.50910 (11)	0.5615 (2)	0.13088 (14)	0.0304 (5)	
C11	0.55669 (11)	0.6412 (2)	0.17671 (15)	0.0343 (5)	
H11	0.5891	0.6194	0.2299	0.041*	
C12	0.55782 (11)	0.7562 (2)	0.14517 (16)	0.0355 (5)	
C13	0.50789 (13)	0.7859 (2)	0.06268 (17)	0.0417 (6)	
H13	0.5073	0.8605	0.0400	0.050*	
C14	0.46131 (13)	0.7060 (2)	0.01705 (16)	0.0412 (6)	
H14	0.4303	0.7270	−0.0374	0.049*	
C15	0.65369 (14)	0.8065 (3)	0.27868 (18)	0.0514 (7)	
H15A	0.6912	0.8598	0.2929	0.062*	
H15B	0.6702	0.7277	0.2782	0.062*	
C16	0.6252 (3)	0.8148 (4)	0.3481 (2)	0.0935 (13)	
H16A	0.5824	0.7771	0.3282	0.140*	
H16B	0.6203	0.8957	0.3603	0.140*	
H16C	0.6547	0.7768	0.4000	0.140*	
C17	0.60749 (14)	0.9541 (2)	0.1600 (2)	0.0490 (7)	
H17A	0.5944	0.9516	0.0973	0.059*	
H17B	0.6532	0.9818	0.1850	0.059*	
C18	0.56346 (19)	1.0404 (3)	0.1815 (3)	0.0720 (10)	
H18A	0.5791	1.0499	0.2434	0.108*	
H18B	0.5185	1.0116	0.1598	0.108*	
H18C	0.5648	1.1145	0.1549	0.108*	

### Atomic displacement parameters (Å^2^)

**Table:**

	*U*^11^	*U*^22^	*U*^33^	*U*^12^	*U*^13^	*U*^23^
S1	0.0310 (4)	0.0404 (4)	0.0444 (4)	−0.0065 (2)	0.0080 (3)	−0.0132 (3)
O1	0.0836 (16)	0.1016 (18)	0.0462 (12)	−0.0473 (14)	0.0318 (12)	−0.0338 (12)
O2	0.0341 (10)	0.0851 (15)	0.0515 (11)	−0.0193 (9)	0.0150 (8)	−0.0287 (10)
O3	0.0664 (15)	0.0437 (13)	0.128 (2)	0.0007 (11)	−0.0253 (15)	−0.0222 (14)
O4	0.102 (3)	0.148 (3)	0.120 (3)	0.026 (2)	−0.002 (2)	0.010 (2)
O5	1.22 (7)	0.36 (2)	1.12 (5)	−0.11 (3)	0.75 (6)	−0.22 (3)
O6	0.0317 (9)	0.0359 (9)	0.0378 (9)	−0.0044 (7)	−0.0004 (7)	0.0086 (7)
N1	0.0283 (10)	0.0372 (11)	0.0262 (10)	−0.0048 (8)	0.0018 (8)	0.0015 (8)
N2	0.0344 (11)	0.0346 (11)	0.0480 (12)	−0.0076 (9)	0.0112 (9)	−0.0070 (9)
C1	0.0255 (11)	0.0394 (13)	0.0366 (12)	−0.0036 (9)	0.0101 (10)	−0.0069 (10)
C2	0.0353 (12)	0.0305 (12)	0.0386 (13)	−0.0035 (9)	0.0150 (10)	−0.0023 (9)
C3	0.0296 (11)	0.0340 (12)	0.0279 (11)	0.0010 (9)	0.0106 (9)	0.0014 (9)
C4	0.0271 (11)	0.0359 (12)	0.0266 (11)	−0.0046 (9)	0.0087 (9)	−0.0020 (9)
C5	0.0355 (13)	0.0324 (12)	0.0380 (13)	−0.0030 (10)	0.0027 (10)	0.0054 (10)
C6	0.0332 (13)	0.0428 (14)	0.0363 (13)	−0.0028 (10)	0.0005 (10)	0.0015 (10)
C7	0.0459 (15)	0.0393 (14)	0.0376 (13)	0.0019 (11)	0.0049 (11)	0.0055 (10)
C8	0.0305 (12)	0.0382 (13)	0.0284 (11)	−0.0007 (9)	0.0066 (9)	0.0026 (9)
C9	0.0278 (12)	0.0349 (12)	0.0304 (12)	−0.0024 (9)	0.0073 (9)	0.0012 (9)
C10	0.0269 (11)	0.0323 (11)	0.0304 (11)	−0.0003 (9)	0.0091 (9)	0.0022 (9)
C11	0.0279 (11)	0.0361 (12)	0.0332 (12)	0.0001 (9)	0.0053 (9)	0.0001 (9)
C12	0.0309 (12)	0.0356 (13)	0.0417 (13)	−0.0022 (10)	0.0157 (10)	−0.0053 (10)
C13	0.0430 (14)	0.0343 (13)	0.0427 (14)	−0.0049 (10)	0.0105 (11)	0.0065 (10)
C14	0.0394 (14)	0.0411 (14)	0.0354 (13)	−0.0014 (10)	0.0056 (11)	0.0078 (10)
C15	0.0443 (16)	0.0477 (16)	0.0514 (16)	−0.0131 (12)	0.0061 (13)	−0.0089 (12)
C16	0.137 (4)	0.091 (3)	0.053 (2)	0.004 (3)	0.038 (2)	−0.004 (2)
C17	0.0440 (15)	0.0393 (14)	0.0621 (17)	−0.0096 (12)	0.0185 (13)	−0.0068 (13)
C18	0.072 (2)	0.0533 (19)	0.089 (3)	0.0109 (17)	0.0290 (19)	−0.0022 (17)

### Geometric parameters (Å, °)

**Table:**

S1—O3	1.426 (2)	C7—H7A	0.9600
S1—O2	1.449 (2)	C7—H7B	0.9600
S1—O1	1.450 (2)	C7—H7C	0.9600
S1—C1	1.783 (2)	C8—C9	1.399 (3)
O4—H16	0.8502	C8—H8	0.9300
O4—H15	0.8505	C9—C14	1.419 (3)
O5—H18	0.8491	C9—C10	1.434 (3)
O5—H17	0.8505	C10—C11	1.369 (3)
O6—C10	1.349 (3)	C11—C12	1.417 (3)
O6—H6	0.8200	C11—H11	0.9300
N1—C8	1.311 (3)	C12—C13	1.427 (3)
N1—C4	1.417 (3)	C13—C14	1.357 (3)
N1—H1	0.8600	C13—H13	0.9300
N2—C12	1.356 (3)	C14—H14	0.9300
N2—C15	1.467 (3)	C15—C16	1.507 (5)
N2—C17	1.470 (3)	C15—H15A	0.9700
C1—C6	1.382 (4)	C15—H15B	0.9700
C1—C2	1.385 (3)	C16—H16A	0.9600
C2—C3	1.398 (3)	C16—H16B	0.9600
C2—H2	0.9300	C16—H16C	0.9600
C3—C4	1.399 (3)	C17—C18	1.506 (4)
C3—C7	1.508 (3)	C17—H17A	0.9700
C4—C5	1.397 (3)	C17—H17B	0.9700
C5—C6	1.380 (3)	C18—H18A	0.9600
C5—H5	0.9300	C18—H18B	0.9600
C6—H6A	0.9300	C18—H18C	0.9600
			
O3—S1—O2	113.29 (17)	C8—C9—C14	118.2 (2)
O3—S1—O1	112.40 (19)	C8—C9—C10	125.4 (2)
O2—S1—O1	111.02 (14)	C14—C9—C10	116.4 (2)
O3—S1—C1	106.26 (12)	O6—C10—C11	123.0 (2)
O2—S1—C1	107.32 (11)	O6—C10—C9	116.08 (19)
O1—S1—C1	106.02 (12)	C11—C10—C9	121.0 (2)
H16—O4—H15	105.3	C10—C11—C12	121.7 (2)
H18—O5—H17	105.4	C10—C11—H11	119.1
C10—O6—H6	109.5	C12—C11—H11	119.1
C8—N1—C4	126.16 (19)	N2—C12—C11	121.1 (2)
C8—N1—H1	116.9	N2—C12—C13	121.4 (2)
C4—N1—H1	116.9	C11—C12—C13	117.5 (2)
C12—N2—C15	121.3 (2)	C14—C13—C12	120.5 (2)
C12—N2—C17	122.2 (2)	C14—C13—H13	119.7
C15—N2—C17	116.4 (2)	C12—C13—H13	119.7
C6—C1—C2	120.0 (2)	C13—C14—C9	122.8 (2)
C6—C1—S1	119.52 (18)	C13—C14—H14	118.6
C2—C1—S1	120.39 (18)	C9—C14—H14	118.6
C1—C2—C3	121.3 (2)	N2—C15—C16	112.9 (3)
C1—C2—H2	119.3	N2—C15—H15A	109.0
C3—C2—H2	119.3	C16—C15—H15A	109.0
C2—C3—C4	117.8 (2)	N2—C15—H15B	109.0
C2—C3—C7	120.1 (2)	C16—C15—H15B	109.0
C4—C3—C7	122.1 (2)	H15A—C15—H15B	107.8
C5—C4—C3	120.8 (2)	C15—C16—H16A	109.5
C5—C4—N1	120.7 (2)	C15—C16—H16B	109.5
C3—C4—N1	118.52 (19)	H16A—C16—H16B	109.5
C6—C5—C4	119.9 (2)	C15—C16—H16C	109.5
C6—C5—H5	120.0	H16A—C16—H16C	109.5
C4—C5—H5	120.0	H16B—C16—H16C	109.5
C5—C6—C1	120.1 (2)	N2—C17—C18	113.9 (3)
C5—C6—H6A	120.0	N2—C17—H17A	108.8
C1—C6—H6A	120.0	C18—C17—H17A	108.8
C3—C7—H7A	109.5	N2—C17—H17B	108.8
C3—C7—H7B	109.5	C18—C17—H17B	108.8
H7A—C7—H7B	109.5	H17A—C17—H17B	107.7
C3—C7—H7C	109.5	C17—C18—H18A	109.5
H7A—C7—H7C	109.5	C17—C18—H18B	109.5
H7B—C7—H7C	109.5	H18A—C18—H18B	109.5
N1—C8—C9	127.1 (2)	C17—C18—H18C	109.5
N1—C8—H8	116.4	H18A—C18—H18C	109.5
C9—C8—H8	116.4	H18B—C18—H18C	109.5
			
O3—S1—C1—C6	159.2 (2)	N1—C8—C9—C10	7.0 (4)
O2—S1—C1—C6	−79.3 (2)	C8—C9—C10—O6	2.7 (3)
O1—S1—C1—C6	39.4 (2)	C14—C9—C10—O6	−176.7 (2)
O3—S1—C1—C2	−17.3 (3)	C8—C9—C10—C11	−177.9 (2)
O2—S1—C1—C2	104.2 (2)	C14—C9—C10—C11	2.7 (3)
O1—S1—C1—C2	−137.1 (2)	O6—C10—C11—C12	178.9 (2)
C6—C1—C2—C3	−2.6 (4)	C9—C10—C11—C12	−0.6 (4)
S1—C1—C2—C3	173.88 (17)	C15—N2—C12—C11	−3.9 (4)
C1—C2—C3—C4	1.2 (3)	C17—N2—C12—C11	179.4 (2)
C1—C2—C3—C7	−176.6 (2)	C15—N2—C12—C13	176.3 (2)
C2—C3—C4—C5	1.4 (3)	C17—N2—C12—C13	−0.4 (4)
C7—C3—C4—C5	179.2 (2)	C10—C11—C12—N2	179.3 (2)
C2—C3—C4—N1	−177.30 (19)	C10—C11—C12—C13	−1.0 (3)
C7—C3—C4—N1	0.5 (3)	N2—C12—C13—C14	179.9 (2)
C8—N1—C4—C5	−12.5 (4)	C11—C12—C13—C14	0.2 (4)
C8—N1—C4—C3	166.3 (2)	C12—C13—C14—C9	2.2 (4)
C3—C4—C5—C6	−2.7 (4)	C8—C9—C14—C13	177.0 (2)
N1—C4—C5—C6	176.0 (2)	C10—C9—C14—C13	−3.6 (4)
C4—C5—C6—C1	1.3 (4)	C12—N2—C15—C16	−78.0 (3)
C2—C1—C6—C5	1.3 (4)	C17—N2—C15—C16	98.9 (3)
S1—C1—C6—C5	−175.2 (2)	C12—N2—C17—C18	85.9 (3)
C4—N1—C8—C9	−179.1 (2)	C15—N2—C17—C18	−91.0 (3)
N1—C8—C9—C14	−173.7 (2)		

### Hydrogen-bond geometry (Å, °)

**Table:**

*D*—H···*A*	*D*—H	H···*A*	*D*···*A*	*D*—H···*A*
N1—H1···O6	0.86	2.10	2.723 (3)	129
N1—H1···O2^i^	0.86	2.58	3.160 (3)	125
O6—H6···O2^ii^	0.82	1.91	2.709 (3)	166
O4—H16···O3^i^	0.85	2.00	2.828 (5)	166
O5—H18···N1^iii^	0.85	2.51	3.35 (3)	174
C2—H2···O3	0.93	2.56	2.915 (4)	103
C5—H5···O1^iv^	0.93	2.55	3.392 (3)	150
C7—H7B···O2^i^	0.96	2.44	3.325 (3)	154
C8—H8···O1^iv^	0.93	2.43	3.353 (3)	172
C15—H15B···O3^ii^	0.97	2.50	3.444 (4)	166
C16—H16C···O5^v^	0.96	2.51	3.42 (3)	160

Symmetry codes: (i) −*x*+1/2, −*y*+1/2, −*z*; (ii) *x*+1/2, −*y*+1/2, *z*+1/2; (iii) *x*, *y*, *z*+1; (iv) −*x*+1/2, *y*+1/2, −*z*−1/2; (v) −*x*+1, *y*, −*z*+3/2.

## References

[bb1] Bruker (2000). *SMART* and *SAINT* Bruker AXS Inc., Madison, Wisconsin, USA.

[bb2] Bu, X. H., Gao, Y. X., Chen, W. & Zhang, R. H. (2001). *J. Rare Earth*, **19**, 70–75.

[bb3] Ranford, J. D., Vittal, J. J. & Wang, Y. M. (1998). *Inorg. Chem.* **37**, 1226–1231. 10.1021/ic970805g 11670327

[bb4] Sheldrick, G. M. (1996). *SADABS* University of Göttingen, Germany.

[bb5] Sheldrick, G. M. (2008). *Acta Cryst.* A**64**, 112–122. 10.1107/S0108767307043930 18156677

